# Dental Caries and Disease

**Published:** 1904-05-07

**Authors:** 


### DENTAL CARIES AND DISEASE.

The truth contained in the saying that "the stomach rules the world" is very generally recognised, but it seems to be forgotten sometimes that defective teeth are potent causes of gastric trouble. Strange as it may seem, we have known an eminent physician treat a patient for indigestion without making any examination of the teeth whatever. It is clear, therefore, that Mr. Kenneth Black [1] is doing a good service in again calling attention to the connection that exists between decayed teeth and disease of other parts, a connection which medical practitioners are sometimes apt to overlook.

With regard to the causes of dental caries, Mr. Black states that the decay is due to the presence in the mouth of free acids and bacteria. The acids dissolve the inorganic salts of the tooth, and the bacteria dissolve the collagenous matrix. The presence of free acids in the mouth may be accounted for in various ways; they may be taken in medicine or food, they may be eructed from the stomach, or the saliva may become acid, but most important of all, the carbo-hydrate food lodged on or between the teeth undergoes acid fermentation from the action of micro-organisms present in the mouth. Normally, however, the saliva is alkaline, and any acids produced in the crevices of the teeth are thus neutralised, and decay prevented. There are two conditions under which the saliva is unable to neutralise the acids produced locally, namely—(1) when it is deficient in alkalinity, and (2) when it is deficient in quantity. As to the former, it is well known that the saliva becomes less alkaline or even acid in any condition of prolonged gastric digestion, a phenomenon which occurs in nearly all cases of dyspepsia. Moreover, the teeth when decayed, further tend to keep up the state of chronic dyspepsia by rendering mastication imperfect. A vicious circle is thus established. In fevers, too, the saliva is less alkaline than normal. It is also deficient in quantity in febrile states, so that the tendency for the teeth to decay under these circumstances is considerable, and the greatest care should be exercised in all fever cases to counteract this tendency by scrupulous attention to oral cleanliness. Any mouth-wash used should be antiseptic, alkaline, and pleasant to use, and Mr. Black suggests, as fulfilling these conditions, a solution of carbolic acid combined with glycerine and sodium bicarbonate. Apart from the evil effects upon digestion, dental caries may be responsible for many ailments, *e.g.* necrosis of the jaws, acute and chronic lymphadenitis, empyema of the maxillary antrum, trismus, and trigeminal neuralgia. To this list Mr. Black adds acute submaxillary cellulitis (Angina Ludovici), actinomycosis, tonsillitis, and certain sapræmic, toxæmic and septicæmic conditions. With regard to submaxillary cellulitis, he has seen two fatal cases in which teeth were the obvious sources of infection. As to the relation between dental caries and tonsillitis, the increased liability to the latter of patients with bad teeth is owing to the septic condition of their mouths.

[1] Guy's Hospital Gazette, April 23.

